# Cohort Study to Determine the Impact of *CYP3A5* Genotype on Tacrolimus Dosing Requirements and Trough Concentrations in Heart Transplant Recipients

**DOI:** 10.1002/phar.70116

**Published:** 2026-02-09

**Authors:** Yanting Wu, Michael T. Eadon, Laurie Schenkelberg, Roopa A. Rao, Todd C. Skaar, Emma M. Tillman, Tyler Shugg

**Affiliations:** ^1^ Division of Clinical Pharmacology, Department of Medicine Indiana University School of Medicine Indianapolis Indiana USA; ^2^ Division of Cardiovascular Medicine, Department of Medicine Indiana University School of Medicine Indianapolis Indiana USA

**Keywords:** CYP3A5, heart transplant, pharmacogenomics, tacrolimus

## Abstract

**Background:**

Tacrolimus is primarily metabolized by Cytochrome P450 (CYP)3A4/5. The Clinical Pharmacogenetics Implementation Consortium recommends increasing the initial dose 1.5‐ to 2‐fold in CYP3A5 expressers to enhance transplant outcomes. Our objective was to investigate the impact of CYP3A5 expresser status on tacrolimus dosing requirements and attainment of target trough concentrations in heart transplant recipients.

**Methods:**

We performed a retrospective cohort analysis of tacrolimus dose, concentration, demographics, CYP3A4/5 genotype, concomitant medications, and biochemical data in heart transplant recipients from December 2020 to August 2023. The primary outcome was the time to first therapeutic trough concentration, compared by CYP3A5 expression status. Secondary outcomes included the tacrolimus dose at target trough and dose‐adjusted tacrolimus trough concentration (C_0_/D). Stepwise multiple regression was performed to account for potential covariates. Moreover, clinical outcomes were assessed at 1‐year post‐transplantation and compared based on CYP3A5 expression status.

**Results:**

Among 33 patients, CYP3A5 expressers (27.3%) required longer to achieve therapeutic trough concentrations (median [Q1, Q3]: expressers: 14 [9.5, 16] days vs. nonexpressers 7.5 [6.0, 11] days; *p* = 0.0073) and required nearly double the tacrolimus dose to reach target concentrations (10 [5.5, 13] mg/day for expressers vs. 5 [3.3, 5.9] mg/day for nonexpressers; *p* = 0.0019). Conversely, the C_0_/D was nearly 2‐fold higher in nonexpressers 2.0 [1.6, 3.4] ng/(mL*mg) than expressers (1.1 [0.83, 1.7] ng/(mL*mg); *p* = 0.0015). Stepwise regression identified route of administration (sublingual vs. oral) at therapeutic trough and initial dose as covariates for all outcomes. All clinical outcomes showed no significant differences based on CYP3A5 expression status, with the exception that poor metabolizers demonstrated higher serum creatinine elevation at 1‐week post‐transplantation (*p* = 0.047).

**Conclusion:**

Our findings highlight the impact of CYP3A5 expresser status on the time needed and dosing requirements to attain tacrolimus therapeutic concentrations in heart transplant recipients, suggesting *CYP3A5*‐guided dosing strategies may improve rapid attainment of therapeutic tacrolimus concentrations.

## 1 Introduction

Tacrolimus is a cornerstone immunosuppressive agent used to prevent rejection in solid organ transplants. Tacrolimus inhibits the phosphatase activity of calcineurin, suppresses T‐cell activation, and reduces the risk of immune‐mediated graft rejection [1]. Despite its clinical efficacy, tacrolimus exhibits a narrow therapeutic index and significant inter‐patient variability in pharmacokinetics [2, 3]. These challenges necessitate therapeutic drug monitoring (TDM), with therapeutic trough concentrations in whole blood serving as a standard practice to monitor its clinical efficacy and mitigate the risk of toxicity [4].

Tacrolimus is primarily metabolized by the cytochrome P450 (CYP) enzymes, CYP3A4 and CYP3A5, in the liver and intestine. Additionally, the efflux transporter ATP Binding Cassette Subfamily B Member 1 (ABCB1) in the intestine also plays an important role by pumping the drug back into the intestinal lumen, further influencing its bioavailability [5]. Genetic polymorphisms in CYP3A genes and variations in ABCB1 expression significantly impact tacrolimus disposition, contributing to considerable variability in dosage requirements and dose‐adjusted trough concentrations among transplant recipients [6, 7, 8]. Specifically, compared to CYP3A5 nonexpressers (i.e., individuals carrying two nonfunctional *CYP3A5* alleles [e.g., *3, *6, or *7]), CYP3A5 expressers (i.e., those who carry at least one functional *CYP3A5* allele) exhibit higher tacrolimus clearance due to enhanced enzyme activity, resulting in lower tacrolimus exposure that can potentially lead to delays in achieving therapeutic trough concentrations [9, 10, 11]. Delays in achieving target trough concentrations and reduced time within the therapeutic window are associated with increased length of stay and worse clinical transplant outcomes [11, 12, 13].

The Clinical Pharmacogenetics Implementation Consortium (CPIC) has established genotype‐based dosing recommendations to optimize tacrolimus exposure. The guidelines propose an initial 1.5‐ to 2‐fold increase in tacrolimus (immediate‐release (IR) form) dose for CYP3A5 expressers (normal metabolizer: NM, intermediate metabolizer: IM), compared to CYP3A5 nonexpresser (poor metabolizer: PM), who receive the standard starting dose [14]. Despite this guidance, critical gaps remain in the implementation of *CYP3A5* genotyping in standard pre‐ and post‐transplant workflows. In addition to CYP3A5 polymorphisms, non‐genetic factors, such as age, drug–drug interactions, delayed graft function, impaired absorption, and hepatic function, may also influence tacrolimus exposure and should be incorporated into dosing algorithms [5]. Studies examining the long‐term clinical impact of CYP3A5 polymorphisms on tacrolimus efficacy and toxicity have not yielded conclusive findings because of the routine use of TDM in adjusting for drug under‐ or over‐exposure [15, 16]. However, delayed achievement of therapeutic trough concentrations is associated with increased length of inpatient hospital stay [9].

Within our institution (Indiana University of Health), *CYP3A4* and *CYP3A5* genotyping are routinely performed in all transplant recipients [17]. However, the *CYP3A5* genotype data have yet to be incorporated into a standard tacrolimus dosing protocol. Our previous investigation evaluated the potential of using *CYP3A5* genotyping as accurate predictors of tacrolimus exposure among kidney transplant patients [9]. In the present study, we extended the investigation to the heart transplant population, aiming to further explore the utility of *CYP3A5* genotype‐guided dosing to expedite the attainment of the first therapeutic trough concentration and other tacrolimus pharmacokinetic‐related outcomes. We conducted a retrospective study in heart transplant recipients with two major objectives: (i) to investigate the associations between CYP3A5 expresser status and the time to and dose requirement at first therapeutic tacrolimus trough concentration, and (ii) to identify additional covariates that may influence these associations.

## 2 Materials and Methods

### 2.1 Clinical Genotyping

For heart transplant recipients, genotyping was conducted either during the listing appointments or at the annual evaluation appointment prior to transplantation as part of standard clinical care. Clinical pharmacogenetic testing was conducted on whole blood samples at the Clinical Laboratory Improvement Amendments–certified, College of American Pathologists–accredited Indiana University Pharmacogenomics Laboratory using a validated custom‐designed OpenArray platform (ThermoFisher, Waltham, MA). The panel included the following star allele‐defining *CYP3A4* and *CYP3A5* variants: CYP3A4*2 (rs55785340); CYP3A4*22 (rs35599367); CYP3A5*3 (rs776746); CYP3A5*6 (rs10264272); and CYP3A5*7 (rs41303343) [18]. Star alleles were categorized into metabolizer phenotypes based on CPIC guidelines [14]. Results, including star allele and genotype‐predicted metabolizer phenotypes, were documented as discrete data, and integrated into the hospital's electronic health record (EHR).

### 2.2 Data Source and Extraction

A retrospective cohort study was conducted using data from the Cerner and Organ Transplant Tracking Record (OTTR) EHR systems at Indiana University (IU) Health. The reporting of this study follows the Strengthening the Reporting of Observational Studies in Epidemiology (STROBE) guideline [19]. The study included all heart transplant recipients at IU Health who received care between December 2020 and August 2023. This timeframe was selected because *CYP3A5* genotype testing was routinely implemented for all heart transplant recipients starting in December 2020, ensuring consistent availability of genotype data for analysis. The study protocol, which included a waiver of informed consent, was approved by the Indiana University Institutional Review Board. The Cerner system provided comprehensive clinical, demographic, and laboratory data, while the OTTR system specifically tracked transplant‐related details. Data elements contained in discrete fields were extracted programmatically, while non‐discrete data were collected manually; all manually collected data was collected by two investigators, with discrepancies adjudicated by the study team. In our study cohort, patients received a standardized, risk‐adapted immunosuppression regimen. Induction therapy was stratified by a calculated immunologic risk score, which took into account race, age, gender, panel reactive antibody, and left ventricular assisted device (LVAD). High‐ or moderate‐risk patients (score ≥ 6) received rabbit anti‐thymocyte globulin (thymoglobulin) with methylprednisolone, while low‐risk patients (score ≤ 5) received basiliximab with methylprednisolone. Tacrolimus initiation was based upon induction type: it was typically delayed until post‐operative days 3 to 5 for patients receiving thymoglobulin due to pending renal recovery but started promptly after surgery for those receiving basiliximab once renal function was stable. But the starting of tacrolimus dose was also evaluated case by case, based on patients' clinical presentation. The standard initial oral dose was 1–2 mg twice daily. Dosing was adjusted to achieve protocol‐defined target trough concentration (8–12 ng/mL). For patients requiring sublingual (SL) administration, the protocol mandated a 50% dose reduction from the planned oral dose, with a doubling upon conversion back to oral therapy. All patients received a triple‐drug maintenance regimen of tacrolimus, mycophenolate mofetil (dose‐adjusted for induction and later for age/weight), and a tapering course of prednisone. Universal antifungal prophylaxis for oral thrush was employed, with nystatin as first‐line therapy and clotrimazole as an alternative for intolerant patients. Concomitant use of clotrimazole was specifically recorded due to its known potential to inhibit tacrolimus metabolism.

Collected data consisted of demographic and clinical information, tacrolimus (IR) dose, route of administration (i.e., oral or SL) at initial and therapeutic dosing, whole blood concentrations (measured by Chemiluminescent Microparticle Immunoassay methodology on the Alinity CI platform), concomitant medications, and relevant laboratory values. Demographic details encompassed race (self‐reported), ethnicity (self‐reported), age at transplantation, sex (self‐reported and on legal identification), weight (measured at transplantation), and height (for body mass index (BMI) calculation). Clinical data included the indication for transplantation, history of LVAD, liver function at initial tacrolimus dose (evaluated by alkaline phosphatase (ALP), aspartate aminotransferase (AST), alanine aminotransferase (ALT), and total bilirubin level), induction therapy (i.e., rabbit anti‐thymocyte globulin or basiliximab), donor risk assessment (high‐risk vs. low‐risk determined by their history of behaviors (e.g., intravenous drug use) that increase the chance of passing on an undetected infection at the time of organ donation), and days required to transition from parenteral induction therapy to oral tacrolimus (induction therapy days). *CYP3A4* and *CYP3A5* genotypes and predicted phenotypes were also extracted. Since only one study participant (3.0%) carried the CYP3A4*22 allele, the analysis focused on the impact of CYP3A5 expresser status on study outcomes. Drug–drug interactions affecting tacrolimus concentrations were recorded for patients who were co‐prescribed tacrolimus from its initiation until the attainment of the first therapeutic trough. Interactions involving CYP3A4/5 inducers or inhibitors were included in the analysis. Clinical CYP3A4/5 inducers and inhibitors were identified based on the United States Food and Drug Administration (FDA) list [20]. Standard post‐transplant prophylactic agents—including antimicrobials for surgical, viral (cytomegalovirus (CMV)), fungal, and opportunistic (pneumocystis jirovecii pneumonia (PJP)) prophylaxis—were not considered, as they are not recognized by the FDA as clinically important CYP3A5 inducers or inhibitors (with the exception of clotrimazole, which was accounted for in the analysis). Additionally, due to its common role in immunosuppressive therapy and classification as a weak CYP3A inducer [21, 22], corticosteroid dosing was documented from the time of transplantation to the achievement of the target tacrolimus trough concentration. All steroid doses were converted to methylprednisolone equivalents (mg) [23]. Laboratory values influencing tacrolimus exposure, such as creatinine clearance (CrCL, calculated using Cockcroft‐Gault formula), hematocrit, and albumin concentration, were also included in the analysis.

Clinically relevant outcomes were collected over a 1‐year follow‐up period of post‐transplantation. These included the incidence of treated infections (confirmed by microbiology result and new addition of infection treatment), the potential for acute kidney injury (assessed by the change between the maximum serum creatinine [SCr] level within 1 week and 1 year post‐transplant relative to baseline), the median of left ventricular ejection fraction throughout 1 year post‐transplantation, and available assessments of acute cellular rejection with grading obtained via routine clinical biopsies during the 1‐year period. Finally, each patient's health status was systematically assessed at annual follow‐up visits, with data extracted from clinical progress notes on Cerner.

### 2.3 Tacrolimus Pharmacokinetics‐Related Outcome Definitions and Analysis

Whole blood tacrolimus concentrations were tracked from the time of initial administration to evaluate attainment of the target trough concentration, defined as 8–12 ng/mL, regardless the type of induction therapy (i.e., rabbit anti‐thymocyte and basiliximab). The primary outcome, time needed to reach the therapeutic trough concentration, was calculated as the interval (days) between tacrolimus initiation and the first date the target concentration exceeded 8 ng/mL. The secondary outcomes were the tacrolimus dose required to achieve first target trough and dose‐adjusted therapeutic trough concentration (*C*_0_/*D*). The dose required to reach the first target trough was defined as the total daily dose given on the day a patient's tacrolimus trough level first achieved the institutional therapeutic range (e.g., > 8 ng/mL). The corresponding *C*_0_/*D* ratio was calculated using this initial therapeutic trough level (*C*_0_) and the total daily dose (*D*) of tacrolimus that the patient was receiving the first time the trough level was drawn. To confirm that the CYP3A5 expresser status was not used to guide tacrolimus dosing, the initial tacrolimus daily dose was also evaluated. All outcomes were compared between CYP3A5 expressers and nonexpressers, as well as across the three CYP3A5 metabolizer phenotypes: NM, IM, and PM. A sensitivity analysis was conducted by standardizing SL tacrolimus doses to oral equivalents (conversion factor: SL: PO = 1:2) to assess its potential influence on the results.

Pairwise comparisons of primary and secondary outcomes were conducted using the Mann–Whitney test in GraphPad Prism v.10.2.2 (GraphPad Software, La Jolla, CA, USA), with statistical significance set at *p* ≤ 0.05. Furthermore, a post hoc power analysis was conducted for the primary outcome (days needed to reach therapeutic tacrolimus trough concentration), using both parametric and non‐parametric methods.

### 2.4 Covariates Identification and Sensitivity Analysis

To explore other potential factors that might influence the association between CYP3A5 expression status and outcomes, all collected demographic and clinical variables were studied. Due to the concern that race is correlated with *CYP3A5* phenotypes, the effects of potential covariate were assessed in three ways: (i) only CYP3A5 phenotypes but not race, along with other covariates were included in the model (CYP3A5 phenotype‐based model), (ii) only race but not CYP3A5 phenotypes, along with other covariates were evaluated (race‐based model), and (iii) both race and CYP3A5 phenotypes, along with other covariates were analyzed (combination model).

Stepwise linear regression was performed using R (v.4.4.2) to identify significant covariates. Key demographic and clinical covariates (Table 1) were included in the multivariable regression analysis. Continuous variables comprised age, BMI, duration of induction therapy (days), CrCL, albumin level, and total cumulative steroid dose. Binary categorical variables included sex, race, high‐risk donor status (yes/no), and presence of a clinically significant drug–drug interaction (yes/no). Finally, primary heart transplant indication and type of induction treatment were included as nominal categorical variables, each with three levels. The covariates were considered important if the *p* value < 0.1.

**TABLE 1:** Demographic and clinical information stratified by CYP3A5 expression.

	Total (*N* = 33)	CYP3A5 expresser (*N* = 9)	CYP3A5 nonexpresser (*N* = 24)	*p* ^a^
Age at transplantation (years)	51 ± 13	46.9 ± 18.6	50.7 ± 10.7	0.33
BMI (kg/m^2^)	25.9 ± 4.0	25.9 ± 5.0	25.5 ± 2.9	0.99
Sex				0.09
Female	10 (30.3)	5 (50)	5 (50)	
Male	23 (69.7)	4 (17.4)	19 (82.6)	
Race				0.09
African American	10 (30.3)	5 (50)	5 (50)	
White	23 (69.7)	4 (17.4)	19 (82.6)	
Ethnicity				> 0.99
Not Hispanic and Latino	32 (97.0)	9 (28.1)	23 (71.9)	
Hispanic and Latino	1 (3.0)	0	1 (100)	
LVAD				> 0.99
Yes	4 (12.1)	1 (25)	3 (75)	
No	29 (87.9)	8 (27.6)	21 (72.4)	
Cardiac transplant Indication				0.15
Cardiomyopathy	18 (54.5)	6 (33.3)	12 (66.7)	
Ischemic cardiomyopathy	5 (15.2)	0	5 (100)	
Nonischemic cardiomyopathy	13 (39.4)	6 (46.2)	7 (53.8)	
Heart failure	13 (39.4)	3 (23.1)	10 (76.9)	
Congenital defect	2 (6.1)	0	2 (100)	
Induction treatment				0.20
Rabbit anti‐thymocyte globulin	15 (45.5)	5 (33.3)	10 (66.7)	
Basiliximab	17 (51.5)	3 (17.6)	14 (82.3)	
Basiliximab and rabbit anti‐thymocyte globulin	1 (3.0)	1 (100)	0	
Days between induction therapy and tacrolimus initial dosing				0.38
Rabbit anti‐thymocyte globulin	3.1 ± 1.1	2.3 ± 0.5	3.6 ± 1.1	
Basiliximab	3.6 ± 2.2	4.7 ± 2.1	3.4 ± 2.2	
Basiliximab and rabbit anti‐thymocyte globulin	6.0	NA	6.0	
Induction therapy days^b^	3.9 (1.7)	3 (1)	4.3 (1.8)	0.05
High‐risk donors	8 (24.2)	1 (12.5)	7 (87.5)	0.40
Drug–drug interactions (DDIs)^c^	5 (15.2)	1 (20)	4 (80)	> 0.99
Liver function				
ALP (units/L)	67.4 ± 31.3	49.6 ± 9.4	77.4 ± 36.2	0.011
ALT (units/L)	47.0 ± 64.9	52.2 ± 58.4	33.4 ± 32.4	0.46
AST (units/L)	78.6 ± 89.0	107.1 ± 132.1	57.7 ± 44.6	0.51
Total Bilirubin (mg/dL)	0.9 ± 0.5	1.0 ± 0.6	0.9 ± 0.4	0.82
CrCL (mL/min)	64.3 ± 24.6	60.0 ± 10.7	66.0 ± 28.2	0.54
Hematocrit (%)	26.7 ± 4.4	24.7 ± 5.6	27.6 ± 3.6	0.09
Albumin (g/dL)	3.1 ± 0.4	3.3 ± 0.6	3.1 ± 0.4	0.24
Total steroid dose (mg)^d^	2114.4 ± 1510.5	2566.3 ± 1955.6	1944.9 ± 1316.9	0.30

*Note:* The descriptive data for continuous variables (age, body mass index (BMI)), post operation day, days between induction therapy and tacrolimus initial dosing, liver function (evaluated by alkaline phosphatase (ALP), aspartate aminotransferase (AST), alanine aminotransferase (AST), and total bilirubin level, creatinine clearance (CrCL), hematocrit, albumin, and total steroid dose) are shown as mean ± standard deviation. For categorical variables (sex, race, ethnicity, left ventricular assisted device placement (LVAD), indication, type induction treatment, high‐risk donors, and drug–drug interactions), data are shown as number of patients with percentage reported in parenthesis. In our study cohort, CYP3A5 expressers have alleles: *1/*1 (*N* = 3), *1/*3 (*N* = 5), *1/*6 (*N* = 1); CYP3A5 nonexpresser have alleles: *3/*3 (*N* = 22), *3/*6 (*N* = 1), *3/*7 (*N* = 1). The percentages in parentheses for the ‘Expressers’ and ‘Nonexpressers’ columns represent the row percentage (i.e., the proportion of patients with a given condition who were expressers or nonexpressers). The ‘Total’ column shows the number and percentage of the entire cohort with each condition.

^a^ *p* values were computed to compare differences between CYP3A5 expressers and nonexpressers. *p* values of continuous variables (age, BMI, hematocrit, albumin, CrCl, and total steroid dose) were computed by unpaired *t*‐test. *p* values of categorical variables (sex, race, indication, induction treatment, high‐risk donors, and DDIs) were obtained by chi‐square test (or Fisher's exact test for expected cell counts of < 5).

^b^ Induction therapy days were defined as the days during which patients received parenteral induction therapy, prior to transitioning to oral medication.

^c^ All the co‐prescribed medications from the initiation of tacrolimus dosing to the attainment of therapeutic trough were evaluated. One CYP3A5 expresser was on fluconazole (moderate CYP3A4 inhibitor) 200 mg, 400 mg, and clotrimazole (strong CYP3A4 inhibitor) 10 mg; four CYP3A5 nonexpressers were on amiodarone (weak CYP3A4 inhibitor) 200 mg during tacrolimus treatment. Drug–drug interaction was treated as a binary categorical variable (Yes/No).

^d^ Total steroid dose was calculated for each patient from initiation of tacrolimus to the attainment of therapeutic trough concentration. All steroid doses were converted to a methylprednisolone equivalent dose, and the average standard deviation of total steroid dose was computed from study cohort (*N* = 33).

To select the best model for each study outcome, stepwise regression was combined with 10‐fold cross‐validation. The lmStepAIC method on R was used to systematically determine the most significant predictors by iteratively adding or removing variables based on the Akaike Information Criterion (AIC). Additionally, key assumptions for stepwise regression were evaluated. Linearity was assessed through residual plots, while independence of errors was tested using the Durbin‐Watson test. Multicollinearity was examined using Variance Inflation Factors (VIF), with values greater than 10 indicating serious multicollinearity. Residual normality was checked with Q‐Q plots and the Shapiro–Wilk test, and homoscedasticity was assessed using the Breusch‐Pagan test.

## 3 Results

### 3.1 Cohort Demographic and Clinical Variables

Thirty‐three patients aged 51 ± 13 years (mean ± standard deviation [SD]) at transplantation and a mean BMI of 25.9 ± 4.0 kg/m^2^ were included in the study (Table 1). The majority of patients were male (69.7%) and White (69.7%). Cardiomyopathy was the most common indication for heart transplantation (54.5%), predominantly nonischemic cardiomyopathy (39.4% of all patients), followed by heart failure (39.4%) and congenital defect (6.1%). Induction therapy included rabbit anti‐thymocyte globulin (45.5%) or basiliximab (51.5%), with one patient receiving a combination of both. After induction therapy, most heart transplant recipients received 0.5 to 1 mg of tacrolimus IR form by mouth twice daily, but alterations in initial dose or route were made based on factors like age, weight, donor risk assessment, liver and kidney function. All patients were transitioned to tacrolimus following a mean induction therapy duration of 3.9 ± 1.7 days. Renal function, as measured by CrCL, averaged 64.3 ± 24.6 mL/min. Other laboratory measures included a mean hematocrit of 26.7% ± 4.4% and serum albumin of 3.1 ± 0.4 g/dL. Total steroid doses varied widely among patients, with a mean ± SD of 2114.4 ± 1510.5 mg (27.9 ± 20.1 mg/kg), or 194.05 ± 216.01 mg per day, from post‐transplantation until attainment of the tacrolimus target therapeutic trough concentration. Drugs that might affect tacrolimus exposure were recorded in five patients (15.2%). The study cohort consisted of nine CYP3A5 expressers (three NMs and six IMs) and 24 nonexpressers (PMs). Most demographic and clinical data were not statistically significant between CYP3A5 expressers and nonexpressers (Table 1).

### 3.2 Assessment of Study Outcomes by Stratified by CYP3A5 Expression Status

**FIGURE 1:**
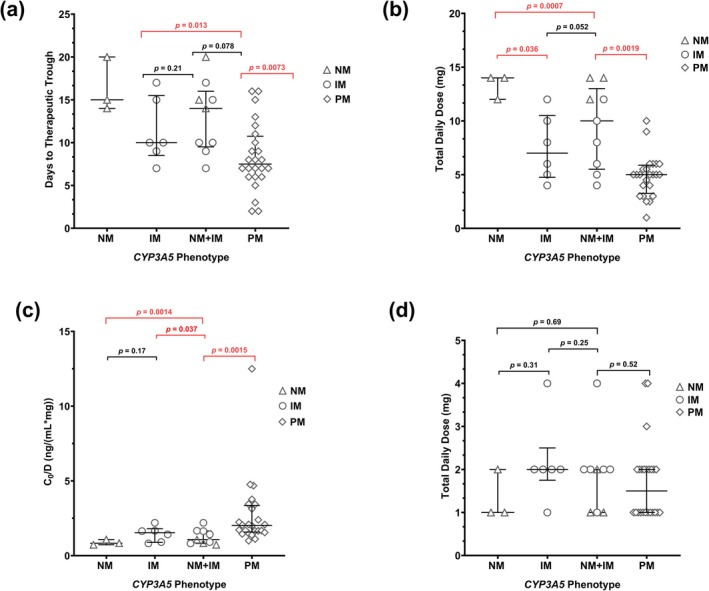
Tacrolimus pharmacokinetic‐related outcomes stratified by CYP3A5 Expression Status: (a) days needed to reach tacrolimus therapeutic trough concentration, (b) daily tacrolimus dose requirement at target trough concentration, (c) dose‐adjusted trough concentration (*C*_0_/*D*), and (d) initial daily tacrolimus dose, stratified by different *CYP3A5* phenotypes. The box plot displays individual values (*N* = 33) with median and interquartile range (first and third quartiles) shown as black lines among different *CYP3A5* phenotypes. The comparison between two groups (expressers vs. nonexpressers) was performed by the Mann–Whitney test, and the statistical significance (highlighted in red) was determined if *p* ≤ 0.05. IM, Intermediate metabolizer; NM, Normal metabolizer; PM, Poor metabolizer. CYP3A5 expresser (*N* = 9): NM + IM, CYP3A5 nonexpresser (*N* = 24): PM.

CYP3A5 expressers required a significantly longer time to reach target trough concentrations compared to nonexpressers [median (Q1, Q3): 14 (9.5, 16) days vs. 7.5 (6.0, 11) days, respectively; *p* = 0.0073] (Figure 1a). A post hoc power analysis confirmed that the study had greater than 90% power to detect this difference by both parametric and non‐parametric methods. In addition, CYP3A5 expressers required double the daily tacrolimus doses to reach first target trough concentrations [10 (5.5, 13) mg/day for expressers vs. 5 (3.3, 5.9) mg/day for nonexpressers; *p* = 0.0019] (Figure 1b). Conversely, CYP3A5 nonexpressers maintained nearly 2‐times higher C_0_/D than expressers [2.0 (1.6, 3.4) ng/(mL*mg) for nonexpressers vs. 1.1 (0.83, 1.7) ng/(mL*mg) for expressers; *p* = 0.0015] (Figure 1c). Because genotype was not incorporated into the dosing algorithm of these 33 patients, no significant difference was observed in the initial tacrolimus dose between the two groups (*p* = 0.52) (Figure 1d). Detailed comparisons among the different CYP3A5 phenotypes are presented in Figure 1. To evaluate the impact of formulation (i.e., SL vs. oral), all SL doses were converted to their oral equivalents. Data incorporating this conversion are shown in Figure S1, and overall trends in the study outcomes were similar, with the most pronounced differences observed between the IM and PM groups.

A comparison of clinical outcomes between CYP3A5 expressers and nonexpressers (Table 2) revealed no statistically significant differences for several key measures, including health status assessment at annual follow‐up, newly acquired infections post‐transplantation, maximum SCr elevation at 1 year post‐transplantation, left ventricular ejection fraction, and acute cellular rejection (*p* values ranging from 0.13 to > 0.99). However, a marginally significant difference was observed, with nonexpressers exhibiting a higher elevation in SCr at 1 week post‐transplantation (*p* = 0.047).

**TABLE 2:** Collected clinical outcomes over 1 year post transplantation stratified by CYP3A5 expression status.

	CYP3A5 expresser (*N* = 9)	CYP3A5 nonexpresser (*N* = 24)	*p* ^a^
Health status at annual follow‐up			0.16
Healthy/absence of poor health outcomes	9	18	
Poor health outcomes	0	6	
Deceased	0	2	
Edema, shortness of breath, increased blood pressure or heart rate	0	3	
Rejection due to nonadherence to tacrolimus	0	1	
New infection	0	3	0.56
Serum creatine Elevation (mg/dL)			
Maximum in 1 week post transplantation/baseline	1.5 ± 0.6	2.1 ± 0.9	**0.047**
Maximum in 1 year post transplantation/baseline	1.9 ± 0.8	1.5 ± 0.8	0.13
Left ventricular ejection fraction (%)	54.5 ± 10.8	59.2 ± 3.7	0.29
Acute cellular rejection (Grade)			> 0.99
0 (no evidence for rejection)	7	17	
1 (mild rejection)	2	7	

*Note:* The descriptive data for serum creatine elevation and left ventricular rejection fraction are shown as mean ± standard deviation. For outcomes at annual follow‐up, infection, acute cellular rejection data are shown as number of patients. In our study cohort, CYP3A5 expressers have alleles: *1/*1 (*N* = 3), *1/*3 (*N* = 5), *1/*6 (*N* = 1); CYP3A5 nonexpresser have alleles: *3/*3 (*N* = 22), *3/*6 (*N* = 1), *3/*7 (*N* = 1). Bold value indicates *p* < 0.05.

^a^ *p* values were computed to compare differences between CYP3A5 expressers and nonexpressers. *p* values for serum creatinine elevation and left ventricular rejection fraction were computed by unpaired *t*‐test. *p* values for outcomes at annual follow‐up, infection, and acute cellular rejection were obtained by chi‐square test (or Fisher's exact test for expected cell counts of < 5).

### 3.3 Covariate Identification and Assessment

The underlying assumptions of stepwise regression models were evaluated to ensure compliance with statistical criteria (Table S1, Figure S2). The identified covariates influencing the study outcomes with CYP3A5 phenotype‐based model are summarized in Table 3. For the time required to achieve target tacrolimus trough concentration (Table 3a), CYP3A5 expresser status had the most significant impact, increasing the time to reach the target concentration by 6.61 days (*p* < 0.0001) when including the use of SL tacrolimus at the first target trough dose (coefficient or *β* = −4.42, *p* = 0.011) and initial tacrolimus dose (*β* = −1.63, *p* = 0.025) as significant covariates. Overall, the regression model explained a substantial proportion of variance (Adjusted *R* ^2^ = 0.41). For the dose requirements to achieve first target trough concentration (Table 3b), CYP3A5 expresser status remained the most significant predictor (*p* < 0.00001), with an increase of 4.8 mg/day on tacrolimus dosing requirement. Other significant covariates in the model were SL tacrolimus formulation used at the first target trough dose (*β* = −2.48, *p* = 0.016), the initial tacrolimus dose (*β* = −1.22, *p* = 0.0041), and age at transplantation (*β* = −0.071, *p* = 0.019). The regression model also demonstrated strong explanatory power (Adjusted *R* ^2^ = 0.67). For the dose‐adjusted trough concentration (Table 3c), CYP3A5 expresser status was also significantly associated with 1.46 ng/(mL*mg) lower therapeutic concentration per dose (*p* = 0.032). Induction therapy with globulin and basiliximab (*β* = −3.88, *p* = 0.032) and SL formulation at trough concentration (*β* = 2.90, *p* = 0.0015) appeared to be the strongest predictors with BMI and high‐risk donor also included in the model. The model explained a considerable portion of variability (Adjusted *R* ^2^ = 0.57).

**TABLE 3:** CYP3A5 phenotype‐based model: Potential covariates identified from stepwise regression for study outcomes: (a) days needed to reach tacrolimus therapeutic trough concentration, (b) dose required at target trough concentration, and (c) dose‐adjusted therapeutic trough concentration (*C*_0_/*D*) The model incorporated CYP3A5 expresser status, along with other demographic and clinical covariates, but did not include race as a variable.

Predictors	Coefficient (*β*)	SE	*t*	Probability
**(a) Dependent variable: Days required to reach therapeutic tacrolimus trough concentration** **Formula: days ~ CYP3A5 Expresser + SL (dose at trough) + initial dose + HCT**				
None (intercept only)	4.71	4.44	1.06	0.30
CYP3A5 Expresser (Y)	6.61	1.46	4.53	**9.98E−05**
SL (dose at trough, Y)^a^	−4.42	1.62	−2.73	**0.011**
Initial dose (mg)	−1.63	0.69	−2.37	**0.025**
HCT (%)	0.25	0.15	1.71	**0.099**
Residuals (median)	0.32		Adjusted *R*‐squared	0.41
Residual standard error	3.42		*F*‐statistic	6.66
Multiple *R*‐squared	0.49		*p* (*F*‐stats)	**0.00067**
**(b) Dependent variable: Dose required to reach therapeutic tacrolimus trough concentration** **Formula: dose ~ CYP3A5 Expresser + initial dose + SL (dose at trough) + age at transplantation + albumin + steroid + sex**				
None (Intercept only)	2.86	3.95	0.72	0.48
CYP3A5 Expresser (Y)	4.80	0.86	5.61	**7.86E−06**
SL (dose at trough, Y)^a^	−2.48	0.96	−2.57	**0.016**
Initial dose (mg)	−1.22	0.39	−3.16	**0.0041**
Age at transplantation (years)	−0.071	0.028	−2.51	**0.019**
Albumin (g/dL)	1.94	0.94	2.05	**0.05055**
Total steroid dose (mg)^b^	0.00049	0.00025	1.95	**0.06217**
Sex (male)	1.50	0.81	1.85	**0.076**
Residuals (median)	0.17		Adjusted *R*‐squared	0.67
Residual standard error	1.89		*F*‐statistic	10.13
Multiple *R*‐squared	0.74		*p* (*F*‐stats)	**5.78E−06**
**(c) Dependent variable: Dose‐adjusted tacrolimus therapeutic trough concentration (*C***_**0**_ **/*D*)** **Formula: *C***_**0**_ **/*D* ~ SL (dose at trough) + BMI + initial dose + CYP3A5 Expresser + induction therapy + high‐risk donor + SL (initial dose) + age at transplantation + indication**				
None (Intercept only)	3.46	1.92	1.80	0.085
SL (dose at trough, Y)^a^	2.90	0.81	3.60	**0.0015**
BMI (kg/m^2^)	−0.19	0.069	−2.74	**0.012**
Initial dose (mg)	0.73	0.31	2.38	**0.026**
CYP3A5 Expresser (Y)	−1.46	0.64	−2.29	**0.032**
Induction therapy (rabbit anti‐thymocyte globulin and basiliximab)	−3.88	1.70	−2.29	**0.032**
High‐risk donor (Y)	1.31	0.63	2.09	**0.048**
SL (initial dose, Y)^b^	1.45	0.79	1.84	**0.079**
Age at transplantation (years)	0.032	0.021	1.52	0.14
Indication (heart failure)	0.76	0.56	1.36	0.19
Residuals (median)	−0.16		Adjusted R‐squared	0.57
Residual standard error	1.38		*F*‐statistic	5.13
Multiple *R*‐squared	0.69		*p* (*F*‐stats)	**0.00035**

*Note:* Bold value indicates *p* < 0.1.

Abbreviations: HCT, hematocrit; SE, standard error of coefficient; SL, sublingual formulation of tacrolimus; Y, yes, for respective categorical variable.

^a^ For the stepwise linear regression, we considered only the use of sublingual tacrolimus at the time of initial administration [SL (initial dose)] and at the time of therapeutic trough [SL (dose at trough)]. Also, sublingual administration was treated as a categorical variable (yes vs. no). For example, SL (dose at trough, Y) indicates that the sublingual formulation was used at the time the therapeutic trough was reached. We did not account for changes in formulation during the overall course of tacrolimus therapy.

^b^ Total steroid dose was calculated for each patient from initiation of tacrolimus to the attainment of therapeutic trough concentration. All steroid doses were converted to methylprednisolone equivalent dose.

Due to the strong correlation between race and CYP3A5 phenotype, their impact on study outcomes was evaluated separately and in combination to determine the most predictive model. The regression results incorporating race but not CYP3A5 phenotype (race‐based model) are presented in Table S2, and the results including both race and CYP3A5 phenotype are provided in Table S3. Additionally, Table S4 summarizes the comparisons of different covariate inclusion strategies and the results from regression models. A comparison of the models revealed that the inclusion of CYP3A5 phenotype significantly improved the predictive power and model fit over the race‐only model.

For the days to reach therapeutic tacrolimus trough concentration (Table S4a), the model incorporating CYP3A5 phenotype and other covariates demonstrated better predictive power (adjusted *R* ^2^ = 0.41) compared to the model including race and other covariates (adjusted *R* ^2^ = 0.23). Moreover, combining CYP3A5 phenotype and race did not improve model performance beyond using CYP3A5 phenotype (both with adjusted *R* ^2^ = 0.41), suggesting that CYP3A5 status (*β* = 6.61) is the dominant predictor for this outcome. For the dose required at first therapeutic tacrolimus trough concentration (Table S4b), the combined model (CYP3A5 phenotype + race) provided the highest predictive power (adjusted *R* ^2^ = 0.70), with CYP3A5 phenotype (*β* = 3.33) contributing more than race (*β* = −2.54) to the overall variability. The CYP3A5 phenotype model alone explained a good proportion of variabilities (adjusted *R* ^2^ = 0.67) with CYP3A5 phenotype being the most significant predictor (*β* = 4.80). In contrast, the race‐based model identified White race (*β* = −3.24), high‐risk donor status (*β* = −3.85), and albumin levels (*β* = 3.29) as significant predictors, but it had the lowest adjusted *R* ^2^ (0.61). For the dose‐adjusted trough concentration (Table S4c), the CYP3A5 phenotype model and the combined model identified the same covariates and performed similarly (both with adjusted *R* ^2^ = 0.57), while the race‐based model had lower explanatory power (adjusted *R* ^2^ = 0.52). In summary, these findings demonstrate that CYP3A5 phenotype is the strongest predictor of tacrolimus pharmacokinetics‐related outcomes, particularly for time to therapeutic trough concentration and dose‐adjusted trough concentration.

## 4 Discussion

To study the association between CYP3A5 phenotypes and tacrolimus pharmacokinetics‐related outcomes, we conducted a retrospective study, integrating both clinical and pharmacogenomic data from a well‐characterized cohort of heart transplant recipients. Our findings showed that CYP3A5 expressers required nearly 7 additional days (*p* = 0.0073) and double the dosage (*p* = 0.0019) to achieve the first tacrolimus target trough concentrations compared to nonexpressers. In contrast, nonexpressers required nearly double the dose‐adjusted trough concentrations relative to expressers (*p* = 0.0015). Moreover, our analysis confirmed that *CYP3A5* genotype‐guided tacrolimus dosing had not been implemented in this cohort. These results align with our previous investigation in kidney transplantation [9] and findings from existing literature in kidney [10, 24], heart [25, 26], and lung [27, 28] transplantation. The collective evidence highlights the important role of CYP3A5 expression status on tacrolimus metabolism and pharmacokinetic‐related outcomes (i.e., time and dosage needed to reach therapeutic trough and dose‐adjusted therapeutic trough concentration).

In addition to CYP3A5 phenotype, race (self‐reported Black vs. White) was also investigated as a potential predictor of study outcomes. The FDA‐approved tacrolimus drug label states higher starting doses may be needed for African American populations [29], and transplant recipients with African ancestry often exhibit higher clearance and lower dose‐corrected tacrolimus trough concentration [30]. Moreover, studies have indicated that Black individuals, particularly those with unfavorable socioeconomic status, tend to experience poorer transplant clinical outcomes [31, 32]. In our study, race, with and without the inclusion of CYP3A5 expresser status, along with other potential covariates, were explored as predictors for tacrolimus pharmacokinetic‐related outcomes. Race emerged as a significant predictor of tacrolimus dose requirements at the first target trough concentration (*β* = −3.24, *p* = 0.0025) but was not associated with other outcomes (Table S2). Additionally, other factors, including high‐risk donor status (*β* = −3.85, *p* = 0.0014), albumin level (*β* = 3.29, *p* = 0.0094), and drug–drug interactions (*β* = 3.37, *p* = 0.036), were strong contributors to dose requirement at first target trough (Table S2b).

When comparing the CYP3A5 phenotype‐based, race‐based, and combination models (included both race and CYP3A5 phenotype), the race‐based model had the weakest predictive power for all three outcomes, as indicated by the lowest adjusted *R* ^2^. In contrast, the CYP3A5 phenotype‐based model showed the most predictive strength for most study outcomes. Notably, CYP3A5 expression status remained a significant predictor even when both race and CYP3A5 expression were included in the combination model. This finding suggests that the CYP3A5 phenotype is a more accurate predictor than race. The impact of race on tacrolimus exposure is likely due to the population‐specific distribution of *CYP3A5* polymorphisms. The CYP3A5*3 allele is more prevalent in White individuals [33, 34], as also reflected in our study population, where the majority (82.6%) of nonexpressers were White, and 92% (22/24) of them were homozygous for CYP3A5*3.

Moreover, in addition to genetic factors, other potential clinical and demographic covariates were also evaluated in the present study to determine their influence on the association between CYP3A5 expression status and tacrolimus pharmacokinetics‐related study outcomes. We used a structured approach to model building: stepwise regression to select potential covariates associated with the outcomes, followed by 10‐fold cross‐validation to assess the model's generalizability and penalize overfitting. This 2‐step process enhanced the rigor and reliability of our statistical analysis.

The use of SL tacrolimus was associated with a shorter time to therapeutic trough, lower dose to reach first target trough concentrations and higher dose‐adjusted trough concentration (*C*_0_/*D*), consistent with its rapid absorption, reduced first‐pass metabolism, and enhanced bioavailability studied in solid organ transplantation [35]. The initial tacrolimus dose showed a negative correlation with the time and dose required to attain the first therapeutic concentrations and a positive correlation with *C*_0_/*D*. Although the effect of the initial dose was not as strong as CYP3A5 expression status or SL formulation, it may be a modifiable factor that can be optimized to help patients achieve therapeutic levels more quickly, suggesting opportunities for protocol optimization. Age was identified as a covariate that negatively affects dosage requirements. Studies in pediatric and adolescent populations have shown that younger individuals exhibit higher tacrolimus clearance, likely due to their larger liver size relative to body weight and the different maturation of CYP3A4 enzymes during development [5, 27, 36, 37]. Additionally, other non‐genetic factors such as BMI, type of induction therapy, and donor risk were also identified as covariates influencing tacrolimus pharmacokinetic‐related outcomes. These findings collectively suggest that tacrolimus exposure is influenced by multiple factors, emphasizing the importance to integrate both pharmacogenomic data with dynamic clinical parameters for the development of genotype‐guided first tacrolimus dosing in transplant recipients.

Although the association between CYP3A5 polymorphism and tacrolimus pharmacokinetics is well‐studied, the impact of CYP3A5 genetic variation on tacrolimus' long‐term efficacy, toxicity, and clinical outcomes remains inconclusive. In current practice at our institution, parenteral induction therapy (basiliximab and rabbit anti‐thymocyte globulin) is utilized to help heart transplant patients achieve quicker immunosuppression, and afterward tacrolimus remains the key medication in patients' therapy. A rapid dose escalation of tacrolimus in CYP3A5 expressers has been used to address the potential for tacrolimus underexposure in this population. Our study adds value to the growing evidence that *CYP3A5* genotype is an accurate predictor to guide initial tacrolimus dosing and reduce the time for transplant recipients to reach first therapeutic trough concentration, which might ultimately optimize their clinical transplant outcomes. However, patient characteristics and other relevant clinical factors still need to be considered for clinical implementation.

Certain limitations of our study must be considered. First, the relatively small sample size (*N* = 33) may limit the generalizability of our findings to broader transplant populations. Second, although efforts were made to account for potential covariates, some other potential factors (i.e., inflammation status/biomarkers [38, 39], gut microbiome composition [40, 41]) that were not collected may influence tacrolimus pharmacokinetics. Third, this retrospective study relies on EHR data that is inherently subject to documentation inconsistency; although we are not aware of systematic data missing, the possibility of unmeasured or unrecorded variables cannot be excluded. Moreover, despite institutional protocols, the precise timing of tacrolimus trough draws may have varied in practice, introducing a potential source of pharmacokinetic measurement variability. Additionally, our study focused on tacrolimus pharmacokinetics‐related outcomes. The analysis of the association between CYP3A5 expresser status and clinical transplant outcomes, such as rejection or graft function, was limited to the first‐year post‐transplantation and does not address long‐term effects.

## 5 Conclusion

Our findings provide further evidence that CYP3A5 expression status significantly influences tacrolimus pharmacokinetics in heart transplant recipients. CYP3A5 expressers exhibit prolonged time to reach first target concentrations and require higher daily tacrolimus doses, while nonexpressers show higher dose‐adjusted trough concentrations. *CYP3A5* genotype‐guided initial dosing may be promising to enhance individualized dosing strategies and improve length of stay. Future studies with larger, diverse cohorts and prospective validation are still warranted to refine genotype‐guided tacrolimus dosing algorithms in transplant care.

## Author Contributions

**Yanting Wu:** conceptualization, methodology, validation, formal analysis, investigation, data curation, writing – original draft, writing – review and editing, visualization. **Michael T. Eadon:** conceptualization, methodology, validation, writing – review and editing, funding acquisition. **Laurie Schenkelberg:** validation, methodology, writing – review and editing. **Roopa A. Rao:** validation, methodology, writing – review and editing. **Todd C. Skaar:** conceptualization, methodology, validation, funding acquisition, writing – review and editing. **Emma M. Tillman:** conceptualization, funding acquisition, methodology, validation, writing – review and editing. **Tyler Shugg:** conceptualization, methodology, validation, investigation, visualization, writing – review and editing, formal analysis, project administration, data curation, supervision, funding acquisition.

## Funding

M.T.E., T.C.S., E.M.T., and T.S. were supported by intramural funding as part of Indiana University's Precision Health Initiative. Y.W. fellowship was supported by the National Institutes of Health/National Institute of General Medical Sciences (NIH/NIGMS) (Grant T32‐GM008425). M.T.E. was supported by the National Institute of Health/National Center for Complementary and Integrative Health (NIH/NCCIH) (Grant R01AT011463). T.S. was supported by the National Institutes of Health/National Institute of General Medical Sciences (NIH/NIGMS) (Grant K23GM147805).

## Conflicts of Interest

The authors declare no conflicts of interest.

## Supporting information


**Table S1:** Assessment of underlying assumptions for stepwise linear regression model.
**Table S2:** Race‐based model: potential covariates identified from stepwise regression for study outcomes: (a) days needed to reach tacrolimus therapeutic trough concentration, (b) dose required at target trough concentration, and (c) dose‐adjusted therapeutic trough concentration.
**Table S3:** Combination model: potential covariates identified from stepwise regression for study outcomes: (a) days needed to reach tacrolimus therapeutic trough concentration, (b) dose required at target trough concentration, and (c) dose‐adjusted therapeutic trough concentration.
**Table S4:** Comparison of significant predictors and coefficient identified from stepwise linear regression when incorporating different covariates for outcomes (a) days needed to reach tacrolimus therapeutic trough concentration, (b) dose required at target trough concentration, and (c) dose‐adjusted therapeutic trough concentration.
**Figure S1:** Tacrolimus pharmacokinetic‐related outcomes stratified by CYP3A5 Expression Status, with sublingual dose converted to equivalent oral dose (SL: PO = 2:1): (a) daily tacrolimus dose requirement at target trough concentration, (b) dose‐adjusted trough concentration (C_0_/D), and (c) initial daily tacrolimus dose, stratified by different *CYP3A5* phenotypes.
**Figure S2:** Assessment of linearity and normality of residuals of stepwise linear regression.

## Data Availability

The authors declare that all the summary data supporting the findings of this study are contained within the paper and its supplemental material. Any individual data can be available and will be shared upon request to the contact author.
