# Supplementary material for: Cohort Study to Determine the Impact of CYP3A5 Genotype on Tacrolimus Dosing Requirements and Trough Concentrations in Heart Transplant Recipients
Source: Pharmacotherapy. 2026 Feb 9;46(3):e70116. doi: 10.1002/phar.70116 (PMC12884575; doi:10.1002/phar.70116)
Supplement: Supplementary file 1 — Table S1: Assessment of underlying assumptions for stepwise linear regression model. Table S2: Race‐based model: potential covariates identified from stepwise regression for study outcomes: (a) days needed to reach tacrolimus therapeutic trough concentration, (b) dose required at target trough concentration, and (c) dose‐adjusted therapeutic trough concentration. Table S3: Combination model: potential covariates identified from stepwise regression for study outcomes: (a) days needed to reach tacrolimus therapeutic trough concentration, (b) dose required at target trough concentration, and (c) dose‐adjusted therapeutic trough concentration. Table S4: Comparison of significant predictors and coefficient identified from stepwise linear regression when incorporating different covariates for outcomes (a) days needed to reach tacrolimus therapeutic trough concentration, (b) dose required at target trough concentration, and (c) dose‐adjusted therapeutic trough concentration. Figure S1: Tacrolimus pharmacokinetic‐related outcomes stratified by CYP3A5 Expression Status, with sublingual dose converted to equivalent oral dose (SL: PO = 2:1): (a) daily tacrolimus dose requirement at target trough concentration, (b) dose‐adjusted trough concentration (C0/D), and (c) initial daily tacrolimus dose, stratified by different CYP3A5 phenotypes. Figure S2: Assessment of linearity and normality of residuals of stepwise linear regression. [file PHAR-46-0-s001.docx]

**Table S1. Assessment of underlying assumptions for stepwise linear regression model**

|  | **Outcomes** | | |  |
| --- | --- | --- | --- | --- |
|  | **Days required to reach tacrolimus therapeutic trough concentration** | **Dose required at tacrolimus therapeutic trough concentration** | **Dose-adjusted tacrolimus therapeutic trough concentration** | **Comments** |
| **Durbin-Watson (DW) test** | | | | |
| - DW | 2.26 | 2.06 | 1.86 | All DW values close to 2, indicating no significant autocorrelation found in residuals |
| - p-value | 0.77 | 0.49 | 0.29 |  |
| **Variance Inflation Factor** (**VIF)** | | | | |
| - CYP3A5 expresser | 1.19 | 1.34 | 1.41 | All VIF values are less than 2, indicating low-to-moderate multicollinearity of all predictors |
| - Initial dose | 1.05 | 1.09 | 1.29 |  |
| - Sublingual form of tacrolimus at trough | 1.10 | 1.27 | 1.68 |  |
| - HCT | 1.13 | - | - |  |
| - Sex | - | 1.28 | - |  |
| - Albumin | - | 1.47 | - |  |
| - Total steroid dose | - | 1.26 | - |  |
| - Age at transplantation | - | 1.19 | 1.29 |  |
| - SL from at initial dose | - | - | 1.62 |  |
| - BMI | - | - | 1.27 |  |
| - High risk donor | - | - | 1.26 |  |
| - Induction | - | - | 1.47 |  |
| - Indication | - | - | 1.20 |  |
| **Shapiro-Wilk normality test** | | | | |
| - W | 0.98 | 0.98 | 0.95 | All W values close to 1, indicating nearly perfect normality of residuals |
| - p-value | 0.85 | 0.79 | 0.15 |  |
| **Studentized Breusch-Pagan (BP) test** | | | | |
| - BP | 3.74 | 12.77 | 22.25 | Lower BP values suggest less heteroscedasticity |
| - p-value | 0.44 | 0.078 | 0.0081 |  |

Independence of residuals was tested using the Durbin-Watson test, while multicollinearity was evaluated with Variance Inflation Factors (VIF). Residual normality was checked by the Shapiro-Wilk test, and homoscedasticity (non-constant variance) was assessed using the Breusch-Pagan test.

**Figure S1. Tacrolimus pharmacokinetic-related outcomes stratified by CYP3A5 Expression Status, with sublingual dose converted to equivalent oral dose (SL: PO = 2:1)**: **(a)** daily tacrolimus dose requirement at target trough concentration, **(b)** dose-adjusted trough concentration (C_0_/D), and **(c)** initial daily tacrolimus dose, stratified by different *CYP3A5* phenotypes

The box plot displays individual values (N=33) with median and interquartile range (first and third quartiles) shown as black lines among different *CYP3A5* phenotypes. The comparison between two groups (expressers vs nonexpressers) was performed by the Mann-Whitney test, and the statistical significance (highlighted in red) was determined if *p* ≤ 0.05.

**Abbreviation: NM**: normal metabolizer, **IM**: intermediate metabolizer, **PM**: poor metabolizer,

**CYP3A5 expresser** (N = 9): NM + IM, **CYP3A5 nonexpresser** (N = 24): PM





**Figure S2: Assessment of linearity and normality of residuals of stepwise linear regression**.

Linearity was evaluated with residual plots in R for the outcomes in CYP3A5 phenotype-based model: **(a)** days needed to reach target tacrolimus concentration, **(b)** and dose required at target trough, **(c)** dose-adjusted trough concentration. Normality was examined with QQ plot in R for outcomes: **(d)** days needed to reach target tacrolimus concentration, **(e)** and dose required at target trough, **(f)** dose-adjusted trough concentration.

| 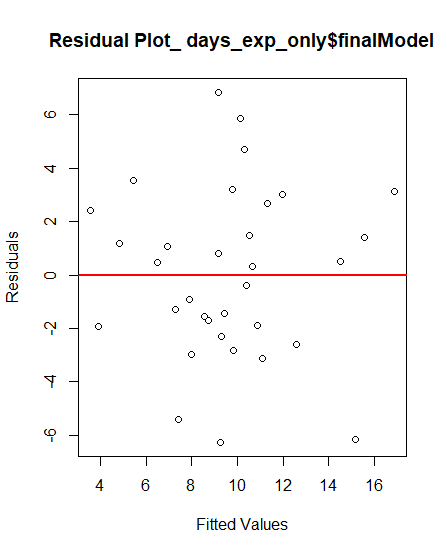  (a) | 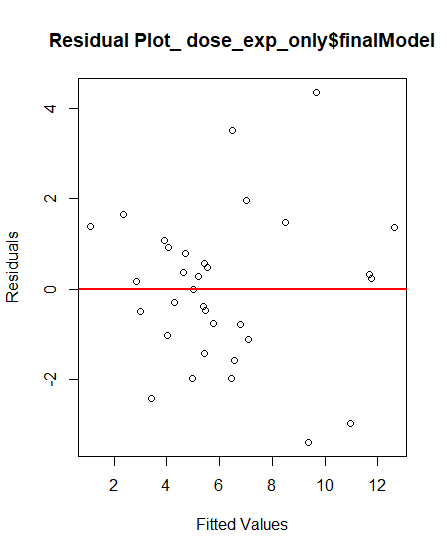  (b) | 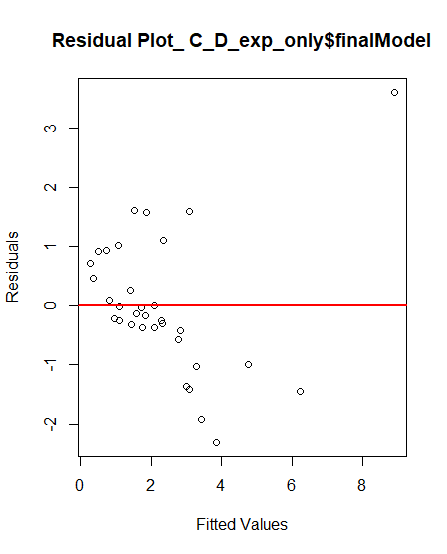  (c) |
| --- | --- | --- |
| 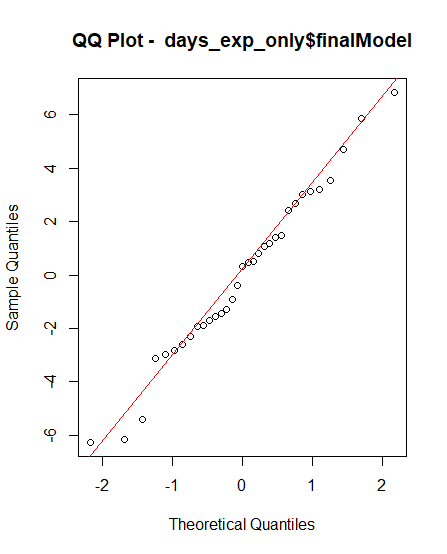  (d) | 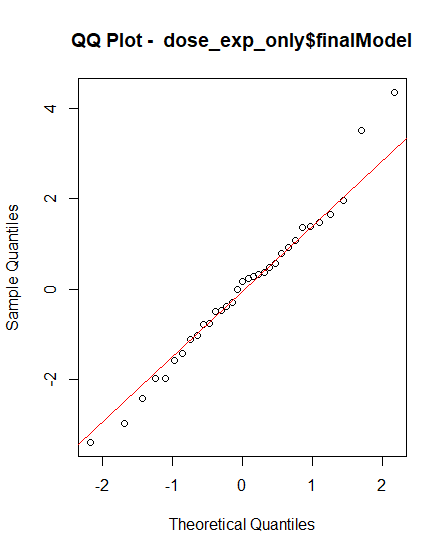  (e) | 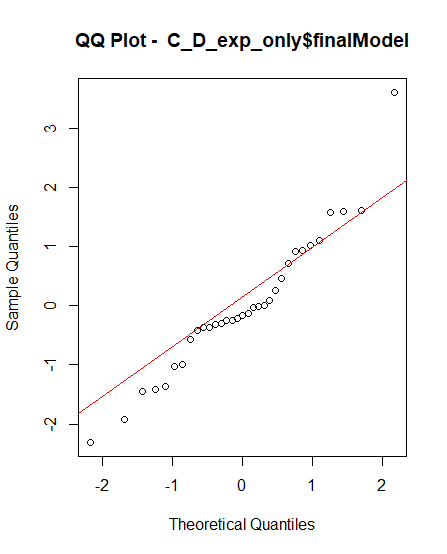  (f) |

**Table S2: Race-based model:** potential covariates identified from stepwise regression for study outcomes: **(a)** days needed to reach tacrolimus therapeutic trough concentration, **(b)** dose required at target trough concentration, and **(c)** dose adjusted therapeutic trough concentration

The model incorporated race, along with other demographic and clinical covariates, but not CYP3A5 phenotypes

| **(a). Dependent variable**: Days required to reach therapeutic tacrolimus trough concentration  **Formula:** days ~ high risk donor + induction therapy + DDI + sex + indication + BMI + albumin + induction therapy + post-op  days + SL (initial dose) | | | | |
| --- | --- | --- | --- | --- |
| **Predictors** | **Coefficient (β)** | **Std error** | **t value** | **Probability** |
| None (Intercept only) | -15.12 | 12.65 | -1.20 | 0.24 |
| **High risk donor (Y)** | -8.59 | 2.66 | -3.23 | **0.0039** |
| **Induction therapy (globulin)** | -5.29 | 1.95 | -2.71 | **0.013** |
| **DDI (Y)** | 7.23 | 2.74 | 2.64 | **0.015** |
| **Sex (male)** | -5.45 | 2.12 | -2.57 | **0.017** |
| **Indication (congenital defect)** | -9.85 | 3.83 | -2.57 | **0.017** |
| **BMI (kg/m^2^)** | 0.54 | 0.25 | 2.15 | **0.043** |
| **Albumin (g/dL)** | 4.86 | 2.33 | 2.09 | **0.049** |
| Induction therapy (globulin and basiliximab) | 8.63 | 4.75 | 1.82 | 0.083 |
| Post-op days | 0.98 | 0.57 | 1.72 | 0.099 |
| SL (initial dose, Y) **^†^** | -3.26 | 2.12 | -1.53 | 0.14 |
| Residuals (median) | -0.27 |  | Adjusted R-squared | 0.23 |
| Residual standard error | 3.92 |  | F-statistic | 1.96 |
| Multiple R-squared | 0.47 |  | p-value (F-stats) | 0.091 |
|  | | | | |
| **(b). Dependent variable**: Dose required to reach therapeutic tacrolimus trough concentration  **Formula**: dose ~ high risk donor + race + initial dose + albumin + SL (initial dose) + post-op days + DDI + indication + age at  transplantation + Induction therapy + BMI | | | | |
| **Predictors** | **Coefficient (β)** | **Std error** | **t value** | **Probability** |
| None (Intercept only) | -1.37 | 6.67 | -0.21 | 0.84 |
| **High risk donor (Y)** | -3.85 | 1.04 | -3.71 | **0.0014** |
| **Race (White)** | -3.24 | 0.94 | -3.45 | **0.0025** |
| **Initial dose (mg)** | -1.57 | 0.52 | -3.01 | **0.0070** |
| **Albumin (g/dL)** | 3.29 | 1.15 | 2.87 | **0.0094** |
| **SL (initial dose, Y) ^†^** | -2.79 | 1.12 | -2.49 | **0.022** |
| **Post-operation days** | 0.63 | 0.26 | 2.36 | **0.028** |
| **DDI (Y)** | 3.37 | 1.50 | 2.25 | **0.036** |
| Indication (heart failure) | -1.68 | 0.85 | -1.97 | 0.062 |
| Age at transplantation (years) | -0.052 | 0.034 | -1.54 | 0.14 |
| Indication (congenital defect) | -3.35 | 2.22 | -1.51 | 0.15 |
| Induction therapy (globulin and basiliximab) | 3.24 | 2.65 | 1.23 | 0.23 |
| BMI (kg/m^2^) | 0.16 | 0.13 | 1.16 | 0.26 |
| Residuals (median) | 0.23 |  | Adjusted R-squared | 0.61 |
| Residual standard error | 2.06 |  | F-statistic | 5.11 |
| Multiple R-squared | 0.75 |  | p-value (F-stats) | 0.00069 |
|  |  |  |  |  |
| **(c). Dependent variable**: Dose adjusted tacrolimus therapeutic trough concentration (C_0_/D)  **Formula**: C_0_/D ~ SL (dose at trough) + BMI + initial dose + SL (initial dose) + + high risk donor + induction therapy + age at transplantation + HCT + indication | | | | |
| **Predictors** | **Coefficient (β)** | **Std error** | **t value** | **Probability** |
| None (Intercept only) | 0.11 | 2.54 | 0.042 | 0.97 |
| **SL (dose at trough, Y) ^†^** | 2.14 | 0.77 | 2.77 | **0.011** |
| **BMI (kg/m^2^)** | -0.18 | 0.073 | -2.52 | **0.019** |
| **Initial dose (mg)** | 0.79 | 0.32 | 2.43 | **0.023** |
| **SL (initial dose, Y) ^†^** | 1.88 | 0.79 | 2.38 | **0.026** |
| **High risk donor (Y)** | 1.50 | 0.66 | 2.29 | **0.031** |
| Induction therapy (globulin and basiliximab) | -3.23 | 1.78 | -1.81 | 0.083 |
| Age at transplantation | 0.037 | 0.022 | 1.67 | 0.11 |
| HCT | 0.092 | 0.061 | 1.49 | 0.15 |
| Indication (heart failure) | 0.79 | 0.59 | 1.34 | 0.19 |
| Residuals (median) | -0.076 |  | Adjusted R-squared | 0.52 |
| Residual standard error | 1.46 |  | F-statistic | 4.84 |
| Multiple R-squared | 0.65 |  | p-value (F-stats) | 0.0011 |
|  |  |  |  |  |

**Abbreviation**: **Std error**: standard error of coefficient, **SL**: sublingual formulation of tacrolimus, **HCT**: hematocrit, **Y**: yes, for respective categorical variable

†. For the stepwise linear regression, we considered only the use of sublingual tacrolimus at the time of initial administration [SL (initial dose)] and at the time of therapeutic trough [SL (dose at trough)]. Also, sublingual administration was treated as a categorical variable (yes vs. no). For example, SL (dose at trough, Y) indicates that the sublingual formulation was used at the time the therapeutic trough was reached. We did not account for changes in formulation during the overall course of tacrolimus therapy.

**Table S3**: **Combination model**: potential covariates identified from stepwise regression for study outcomes**: (a)** days needed to reach tacrolimus therapeutic trough concentration, **(b)** dose required at target trough concentration, and **(c)** dose adjusted therapeutic trough concentration

The model incorporated both CYP3A5 expresser status and race, along with various demographic and clinical covariates.

| **(a). Dependent variable**: Days required to reach therapeutic tacrolimus trough concentration  **Formula:** days ~ CYP3A5 Expresser + SL (dose at trough) + initial dose + HCT | | | | |
| --- | --- | --- | --- | --- |
| **Predictors** | **Coefficient (β)** | **Std error** | **t value** | **Probability** |
| None (intercept only) | 4.71 | 4.44 | 1.06 | 0.30 |
| **CYP3A5 Expresser (Y)** | 6.61 | 1.46 | 4.53 | **9.98E-05** |
| **SL (dose at trough, Y) ^†^** | -4.42 | 1.62 | -2.73 | **0.011** |
| **Initial dose (mg)** | -1.63 | 0.69 | -2.37 | **0.025** |
| HCT (%) | 0.25 | 0.15 | 1.71 | 0.099 |
| Residuals (median) | 0.32 |  | Adjusted R-squared | 0.41 |
| Residual standard error | 3.42 |  | F-statistic | 6.66 |
| Multiple R-squared | 0.49 |  | p-value (F-stats) | 0.00067 |
|  | | | | |
| **(b). Dependent variable**: Dose required to reach therapeutic tacrolimus trough concentration  **Formula**: dose ~ *CYP3A5* Expresser + initial dose + race + age at transplantation + albumin + sex + SL (initial dose) + high risk donor | | | | |
| **Predictors** | **Coefficient (β)** | **Std error** | **t value** | **Probability** |
| **None (Intercept only)** | 6.79 | 3.25 | 2.09 | **0.047** |
| **CYP3A5 Expresser (Y)** | 3.33 | 0.81 | 4.11 | **0.00040** |
| **Initial dose (mg)** | -1.55 | 0.38 | -4.04 | **0.00048** |
| **Race (White)** | -2.54 | 0.80 | -3.18 | **0.0040** |
| **Age at transplantation (years)** | -0.062 | 0.026 | -2.33 | **0.029** |
| **Albumin (g/dL)** | 1.73 | 0.82 | 2.11 | **0.046** |
| Sex (male) | 1.74 | 0.89 | 1.97 | 0.061 |
| SL (initial dose, Y)**^†^** | -1.45 | 0.93 | -1.57 | 0.13 |
| High risk donor (Y) | -1.30 | 0.86 | -1.51 | 0.14 |
| Residuals (median) | -0.16 |  | Adjusted R-squared | 0.70 |
| Residual standard error | 1.79 |  | F-statistic | 10.39 |
| Multiple R-squared | 0.78 |  | p-value (F-stats) | 3.63E-06 |
|  |  |  |  |  |
| **(c). Dependent variable**: Dose adjusted tacrolimus therapeutic trough concentration (C_0_/D)  **Formula**: C_0_/D ~ SL (dose at trough) + BMI + initial dose + *CYP3A5* Expresser + induction therapy + high risk donor + SL (initial dose) + age at transplantation + indication | | | | |
| **Predictors** | **Coefficient (β)** | **Std error** | **t value** | **Probability** |
| None (Intercept only) | 3.46 | 1.92 | 1.80 | 0.084 |
| **SL (dose at trough, Y)^†^** | 2.90 | 0.81 | 3.60 | **0.0015** |
| **BMI (kg/m^2^)** | -0.19 | 0.069 | -2.74 | **0.012** |
| **Initial dose (mg)** | 0.73 | 0.31 | 2.38 | **0.026** |
| **CYP3A5 Expresser (Y)** | -1.46 | 0.64 | -2.29 | **0.032** |
| **Induction therapy (globulin and basiliximab)** | -3.88 | 1.70 | -2.29 | **0.032** |
| **High risk donor (Y)** | 1.31 | 0.63 | 2.09 | **0.048** |
| SL (initial dose, Y)**^†^** | 1.45 | 0.79 | 1.84 | 0.079 |
| Age at transplantation (years) | 0.032 | 0.021 | 1.52 | 0.14 |
| Indication (heart failure) | 0.76 | 0.56 | 1.36 | 0.19 |
| Residuals (median) | -0.16 |  | Adjusted R-squared | 0.57 |
| Residual standard error | 1.38 |  | F-statistic | 5.13 |
| Multiple R-squared | 0.69 |  | p-value (F-stats) | 0.00035 |
|  |  |  |  |  |

**Abbreviation**: **Std error**: standard error of coefficient, **SL**: sublingual formulation of tacrolimus, **HCT**: hematocrit, **Y**: yes, for respective categorical variable

†. For the stepwise linear regression, we considered only the use of sublingual tacrolimus at the time of initial administration [SL (initial dose)] and at the time of therapeutic trough [SL (dose at trough)]. Also, sublingual administration was treated as a categorical variable (yes vs. no) For example, SL (dose at trough, Y) indicates that the sublingual formulation was used at the time the therapeutic trough was reached. We did not account for changes in formulation during the overall course of tacrolimus therapy.

**Table S4.** Comparison of significant predictors and coefficient identified from stepwise linear regression when incorporating different covariates for outcomes **(a)** days needed to reach tacrolimus therapeutic trough concentration, **(b)** dose required at target trough concentration, and **(c)** dose adjusted therapeutic trough concentration

| **(a). Dependent variable: Days required to reach therapeutic tacrolimus trough concentration** | | | | | |
| --- | --- | --- | --- | --- | --- |
| **CYP3A5 Phenotype + Others** | | **Race + Others** | | **CYP3A5 Phenotype +Race + Others** | |
| **Significant predictors** | **Coefficient (β)** | **Significant predictors** | **Coefficient (β)** | **Significant predictors** | **Coefficient (β)** |
| CYP3A5 Expresser (Y) | 6.61 | High risk donor (Y) | -8.59 | CYP3A5 Expresser (Y) | 6.61 |
| SL (dose at trough, Y) **^†^** | -4.42 | Induction therapy (globulin) | -5.29 | SL (dose at trough, Y) **^†^** | -4.42 |
| Initial dose (mg) | -1.63 | DDI (Y) | 7.23 | Initial dose (mg) | -1.63 |
|  |  | Sex (male) | -5.45 |  |  |
|  |  | Indication (congenital defect) | -9.85 |  |  |
|  |  | BMI (kg/m^2^) | 0.54 |  |  |
|  |  | Albumin (g/dL) | 4.86 |  |  |
| **Adjusted R^2^: 0.41** |  | **Adjusted R^2^: 0.23** |  | **Adjusted R^2^: 0.41** |  |
| **(b). Dependent variable: Dose required to reach first therapeutic tacrolimus trough concentration** | | | | | |
| **CYP3A5 Phenotype + Others** | | **Race + Others** | | **CYP3A5 Phenotype +Race + Others** | |
| **Significant predictors** | **Coefficient (β)** | **Significant predictors** | **Coefficient (β)** | **Significant predictors** | **Coefficient (β)** |
| CYP3A5 Expresser (Y) | 4.80 | High risk donor (Y) | -3.85 | None (Intercept only) | 6.79 |
| Initial dose (mg) | -1.22 | Race (White) | -3.24 | CYP3A5 Expresser (Y) | 3.33 |
| SL (dose at trough, Y) **^†^** | -2.48 | Initial dose (mg) | -1.57 | Initial dose (mg) | -1.55 |
| Age at transplantation (years) | -0.071 | Albumin (g/dL) | 3.29 | Race (White) | -2.54 |
|  |  | SL (initial dose, Y)^a^ | -2.79 | Age at transplantation (years) | -0.062 |
|  |  | Post-operation days | 0.63 | Albumin (g/dL) | 1.73 |
|  |  | DDI (Y) | 3.37 |  |  |
| **Adjusted R^2^: 0.67** |  | **Adjusted R^2^: 0.61** |  | **Adjusted R^2^: 0.70** |  |
| **(c). Dependent variable: Dose adjusted tacrolimus therapeutic trough concentration (C_0_/D)** | | | | | |
| **CYP3A5 Phenotype + Others** | | **Race + Others** | | **CYP3A5 Phenotype +Race + Others** | |
| **Significant predictors** | **Coefficient (β)** | **Significant predictors** | **Coefficient (β)** | **Significant predictors** | **Coefficient (β)** |
| SL (dose at trough, Y) **^†^** | 2.90 | SL (dose at trough, Y) **^†^** | 2.14 | SL (dose at trough, Y) **^†^** | 2.90 |
| BMI (kg/m^2^) | -0.19 | BMI (kg/m^2^) | -0.18 | BMI (kg/m^2^) | -0.19 |
| Initial dose (mg) | 0.73 | Initial dose (Y) | 0.79 | Initial dose (mg) | 0.73 |
| CYP3A5 Expresser (Y) | -1.46 | SL (initial dose, Y) **^†^** | 1.89 | CYP3A5 Expresser (Y) | -1.46 |
| Induction therapy (globulin and basiliximab) | -3.88 | High risk donor (Y) | 1.50 | Induction therapy (globulin and basiliximab) | -3.88 |
| High risk donor (Y) | 1.31 |  |  | High risk donor (Y) | 1.31 |
| **Adjusted R^2^: 0.57** |  | **Adjusted R^2^: 0.52** |  | **Adjusted R^2^: 0.57** |  |

**Abbreviation**: **SL**: sublingual formulation of tacrolimus, **HCT**: hematocrit, **Y**: yes, for respective categorical variable

†**.** For the stepwise linear regression, we considered only the use of sublingual tacrolimus at the time of initial administration [SL (initial dose)] and at the time of therapeutic trough [SL (dose at trough)]. Also, sublingual administration was treated as a categorical variable (yes vs. no) For example, SL (dose at trough, Y) indicates that the sublingual formulation was used at the time the therapeutic trough was reached. We did not account for changes in formulation during the overall course of tacrolimus therapy.
